# Rapid Remission of Secondary Membranous Glomerulonephritis Due to Syphilis: A Case Report

**DOI:** 10.7759/cureus.50900

**Published:** 2023-12-21

**Authors:** Rajesh N Ganesh, Solyman Hatami, Tanner Walker, Keri Janowiak, Roberto Barrios

**Affiliations:** 1 Pathology and Genomic Medicine, Houston Methodist Hospital, Houston, USA; 2 Nephrology, Texas A&M University School of Engineering Medicine, Houston, USA; 3 Pathology, Texas A&M University School of Engineering Medicine, Houston, USA

**Keywords:** nephrotic syndrome, full-house pattern immunofluorescence, renal biopsy, rapid remission, syphilis, membranous glomerulonephritis

## Abstract

Membranous glomerulonephritis (MGN) is an antibody-mediated autoimmune disease that targets the glomerular basement membrane-podocyte complex, causing defects in the glomerular filtration barrier and resulting in nephrotic syndrome. Management of patients with MGN now relies on identifying the underlying etiology. A 36-year-old female patient, with a recent history of transient vision loss, presented with 11 days of progressive edema and episodes of vomiting, headache, and stomach pain. Evaluation of progressive proteinuria led to a renal biopsy, which showed normal glomerular histology by light microscopy and a full-house pattern of immune-complex deposits by immunofluorescence microscopy. Electron microscopy showing very occasional subepithelial deposits confirmed the diagnosis of MGN. Testing for anti-PLA2R antibody, a biomarker for primary (idiopathic) MGN, was negative by immunohistochemistry and serology. Extensive clinical evaluation and workup led to a rapid plasma reagin (RPR) test for syphilis, which was positive. Treatment was immediately initiated with furosemide, losartan, and weekly intramuscular benzathine penicillin, and within two weeks, the patient’s edema had subsided, and her proteinuria had resolved. The patient remained in clinical remission at 11-month follow-up with good overall health. We emphasize the importance of early diagnosis of syphilis-induced MGN as prompt treatment results in rapid remission of renal disease. In the evaluation of secondary MGN, atypical presentations of syphilis should be considered in the differential diagnosis to ensure the timely initiation of appropriate management.

## Introduction

Syphilis, caused by the spirochete Treponema pallidum, is a predominately sexually transmitted infection with a variable systemic presentation. It is screened for in developed countries with early tests such as venereal disease research laboratory (VDRL) testing and treated appropriately with penicillin, usually early in its course. We report a case of rapid remission of secondary membranous glomerulonephritis (MGN) after appropriate syphilis penicillin treatment. MGN is among the most common causes of nephrotic syndrome in adults. Identification of autoantibodies against phospholipase A2 receptor (PLA2R) in patients with MGN was a significant milestone in the elucidation of disease pathogenesis. The presence of anti-PLA2R antibodies is now used to distinguish primary MGN from secondary causes, allowing for quicker diagnosis and selection of appropriate treatment. Anti-PLA2R antibodies can be detected by immunohistochemistry in formalin-fixed paraffin-embedded tissues, by immunofluorescence in frozen tissues, and by serology. Anti-PLA2R testing remains the standard of practice in diagnosing primary MGN despite reports of significant variability in sensitivity and specificity [1].

Significant challenges remain in the diagnosis of primary MGN and in the identification of underlying causes of secondary MGN. Several new antibody targets have been identified in the pathogenesis of secondary MGN, and further investigation into the etiology of this disease is critical to the advancement of disease management and patient care.

## Case presentation

**Figure 1:**
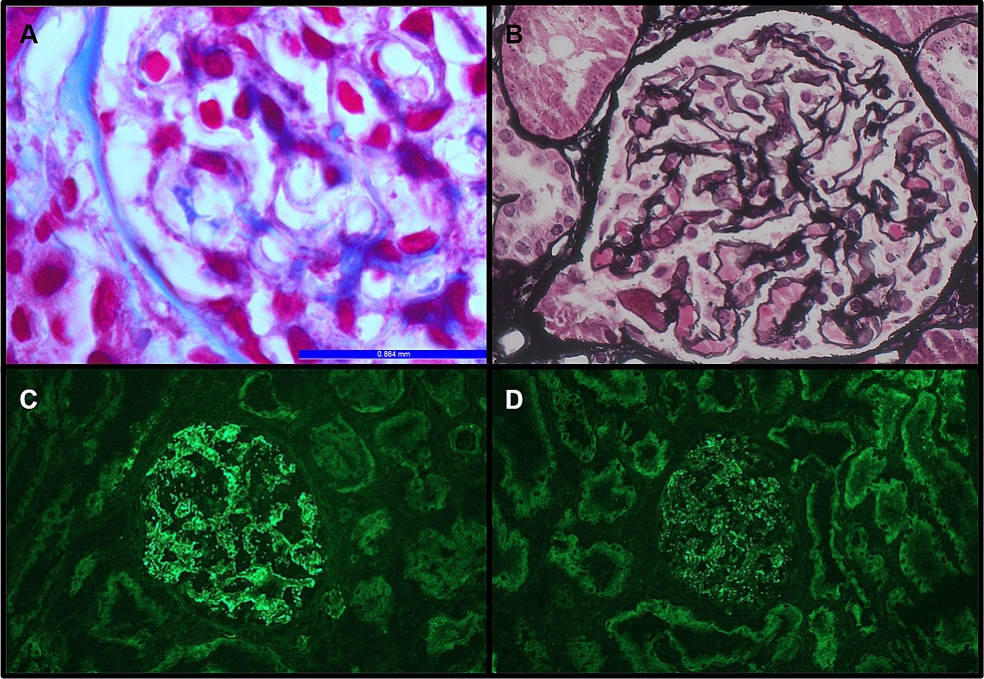
Microscopic Histology and Immunofluorescence A-Section shows glomeruli with normal capillary basement membrane thickness and normal cellularity and tubules within normal limits and subepithelial deposits on the basement membrane, trichrome, x400. B-Section shows glomeruli with normal capillary basement membrane thickness and no evidence of spikes or moth-eaten appearance, Jones methenamine silver stain, x 400. C-Section shows glomeruli with strong coarse granular deposits of IgG along glomerular capillary basement membrane, Fluorescin isothiocyanate stain, 1:20 dilution of IgG with DAKO monoclonal antibody, x200. D-Section shows strong fine granular deposits of C1q along the glomerular capillary basement membrane, Fluorescin isothiocyanate stain, 1:20 dilution of C1q with DAKO monoclonal antibody, x200.

**Figure 2:**
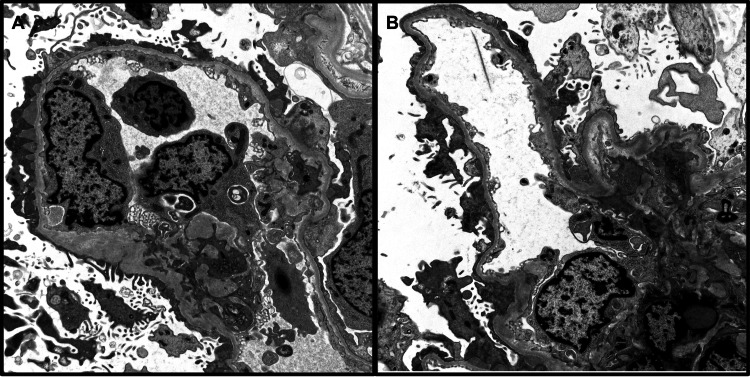
Electron Microscopy A-Section highlights the ultrastructure of the glomerulus with a very occasional subepithelial deposit along the glomerular capillary basement membrane (arrow), uranyl acetate, x3000. B-Section highlights a higher magnification of the discrete subepithelial deposit in the glomerular capillary basement membrane (arrow), uranyl acetate, x8000.

A 36-year-old female patient presented with malaise; a polymorphic, erythematous rash on her lower extremities; and lower extremity edema, which had progressed to ascites and pleural effusion over a period of 11 days. She also had episodes of vomiting, headache, and stomach pain during this time. Five months prior to presentation, she had experienced transient vision loss for which the etiology was never identified. Her initial workup included a normal ophthalmology evaluation, brain MRI without significant abnormality, and negative CSF studies. Laboratory workup revealed full-blown nephrotic syndrome with bland urine sediment; normal serum creatinine, C3, and C4 levels; and a negative autoantibody panel. A kidney biopsy showed normal renal cortical and medullary architecture and renal tubules with prominent intracellular protein resorption droplets. Immunofluorescence microscopy revealed a full-house pattern of immune complex deposition, which includes strong, diffuse, co-dominant IgG, C3, and C1q deposits, as well as IgA, IgM, kappa, and lambda deposits within the glomerular capillary membrane (Figures 1A-1D). Electron microscopy showing glomeruli with occasional subepithelial deposits and focal foot process effacement confirmed the diagnosis of MGN (Figures 2A-2B ).

Additional sexual patient history led to sexually transmitted infection testing and a rapid plasma reagin (RPR) test, which showed an elevated titer in serum and CSF. Testing for parvovirus was not performed given the history and prior testing pointing towards a sexually transmitted infectious source. A diagnosis of MGN secondary to syphilis was made, and the patient was started on weekly benzathine penicillin G, as well as a regimen of furosemide, losartan, and atorvastatin. Within two weeks, her edema and ascites had reduced significantly, her urine albumin was negative, and serum albumin levels had returned to normal (Table 1). She remained in clinical remission and in good health for 11 months.

**Table 1:** Laboratory Parameters

Laboratory parameters (reference range & units)	13 to 16 October 2022	20 October 2022	31 October 2022
Serum glucose (65-99 mg/dL)	115	104	68
Serum creatinine (0.5-0.97 mg/dL)	1.21	1.04	0.84
Sodium (135-146 mmol/L)	137	134	138
Potassium (3.5-5.3 mmol/L)	4.2	3.8	3.5
Calcium (8.6-10.2 mg/dL)	8.3	8.2	9.0
Serum albumin (3.6-5.1 g/dL)	3.3	NA	3.6
Total complement activity (CH50) (38.7-89.9 U/mL)	54.5	NA	NA
Smith antibody	Negative	NA	NA
ANA, DsDNA antibody	Negative	NA	NA
Histone antibody	Negative	NA	NA
C3 complement (90-180 mg/dL)	157	NA	NA
C4 complement (10-40 mg/dL)	38	NA	NA
Urine protein (Negative)	3+	NA	Negative
Urine protein/creatinine ratio (<0.2 ratio)	3.6	2	0.06
CSF VDRL	Negative	NA	NA
CSF PCR for West Nile virus, enterovirus, Epstein Barr virus, Cytomegalovirus, Herpes simplex 1,2 and 6, Human parechovirus, Varicella zoster virus	Negative	NA	NA
CSF Elisa for Borrelia burgorferi antibody (LIV)	Negative	NA	NA
CSF PCR for Meningitis/encephalitis panel (E.coli, H. influenza, N. meningitidis, S. agaliactiae, S. pneumoniae, C. neoformans/gattii	Negative	NA	NA
Syphilis treponema screen with RPR confirmation (reverse algorithm)	Reactive	NA	NA
HIV antigen/antibody fourth generation with reflexes	Negative	NA	NA
Hepatitis acute panel (Hepatitis A IgM, Hepatitis C Ab, Hepatitis B core IgM, Hepatitis B Surface Ag	Negative	NA	NA
Urine culture	No growth	NA	NA

## Discussion

The case highlights the challenges in diagnosis and appropriate management of MGN, particularly in the setting of syphilis, a well-known mimicker of numerous disease states. Our patient had a histologically normal renal cortex and medulla with immunofluorescence showing strong full-house immune complex deposits with co-dominant C1q, a pattern that is characteristically described in systemic lupus erythematosus (SLE). However, several recent studies have identified a large number of non-SLE cases that share the same pattern; in fact, up to 21% of biopsies showing full-house immune complex deposits have been attributed to etiologies other than SLE, and MGN has been identified as a common disease within this sub-group [2-4].

Though it is well-documented that full-house immunofluorescence is not diagnostic of SLE, renal-limited lupus nephritis must still be considered in the differential diagnosis for these findings. Detailed patient history, full serologic workup, and interdisciplinary correlation of clinical findings are necessary to investigate the possibility of underlying SLE thoroughly.

Cases of infection with syphilis alone [5] or co-infection with parvovirus B have been previously associated with full-house immunofluorescence on a kidney biopsy [6]. These reports highlight remarkable variations in patient presentation, laboratory findings, and renal histology. For example, Alvarez et al. [6] reported a patient with features of neurosyphilis and otosyphilis, though the ultrastructural findings were unfortunately unavailable for analysis. Scaperotti et al. [5] reported a case of syphilis-induced MGN in a patient presenting with acute renal failure, cryoglobulinemia, positive anti-cardiolipin antibody, and low complement levels with undetectable C4. This differs significantly from our patient with full-blown nephrotic syndrome, normal eGFR and serum complement levels, and negative auto-immune serology. In addition, the renal biopsy showed histologic changes not observed in our patient, including mesangial hypercellularity, tubular atrophy, and interstitial fibrosis. Despite these differences, both cases share the finding of sparsity in electron-dense deposits within the glomeruli.

One common feature in these and other reports of syphilis-induced membranous nephropathy is a complete resolution of nephrotic syndrome and recovery of renal function despite differences in disease management. The patient reported by Scaperotti et al. [5] was treated with benzathine penicillin, as well as concomitant methylprednisolone and mycophenolate mofetil, likely in response to the autoimmune serology, low C4, and a mesangio-proliferative pattern of glomerular disease. Complete remission was achieved within two months despite concomitant acute renal failure. In our patient and the patient reported by Alvarez et al. [6], treatment was successful without immunosuppressive therapy.

## Conclusions

The incidence of syphilis is increasing worldwide, a trend seen in many diseases associated with immunosuppression and antibiotic resistance. Syphilis-induced membranous nephropathy with full-house immunofluorescence can be misdiagnosed as lupus nephritis, resulting in unnecessary immunosuppression, increasing renal damage, and longer time to patient recovery. Patients in whom full-house immunofluorescence was properly diagnosed as membranous nephropathy secondary to syphilis have shown complete and rapid remission with standard syphilis treatment. This highlights a need for continued documentation of similar cases in the literature in an effort to increase awareness of this unique phenomenon.
